# Predictive Value of Positive Surgical Margins after Radical Prostatectomy for Lymph Node Metastasis in Locally Advanced Prostate Carcinoma

**DOI:** 10.1155/2012/618574

**Published:** 2011-10-03

**Authors:** Wolfgang Otto, Peter Gerber, Wolfgang Rößler, Wolf F. Wieland, Stefan Denzinger

**Affiliations:** Department of Urology, St. Josef Medical Centre, University of Regensburg, Landshuter Straße 65, 93053 Regensburg, Germany

## Abstract

*Introduction*. Suspected locally advanced prostate carcinoma shows lymph node involvement in a high percentage of cases. For a long time, such patients were not radically prostatectomised. In recent years, however, this viewpoint has changed. *Material and Methods*. We analysed a single-centre series of 34 patients with suspected locally advanced prostate cancer to establish predictive parameters for lymph node metastasis. All patients underwent radical prostatectomy between 2007 and 2010. *Results*. Of the 34 patients, 26% showed pathological stage T3a, 59% pT3b, and 15% pT4. Median preoperative PSA level was 25 ng/mL, and five patients had had neoadjuvant antihormonal treatment. Positive margins were found in 76% of patients. Patients without neoadjuvant treatment showed it in 79%, and after preoperative antihormonal treatment the rate was 60%. Positive margins were associated with lymph node involvement in 85% of cases, complete resection was associated only in 50% of cases. *Conclusions*. Positive surgical margins play an important predictive role when estimating lymph node involvement in patients with locally advanced prostate carcinoma. Neoadjuvant antihormonal therapy is associated with a relevant reduction in the rate of positive margins but not with the rate of lymph node metastasis. As such, a combination of antihormonal and surgical treatment should be considered.

## 1. Introduction

According to the European Association of Urology (EAU) guidelines on prostate carcinoma, radical prostatectomy (RP) is the standard treatment for stage T2N0M0 prostate cancer, equivalent to radiation therapy. For locally advanced prostate cancer, recommendations are less concise. In selected patients RP in combination with extended pelvic lymphadenectomy may be feasible. A study by Gontero et al. showed no relevant differences in the rate of comorbidities, only transfusion and lymphocele rate appeared more often compared to T2N0M0 prostate carcinoma. Cancer-specific survival (CSS) was 90% for T3-4, N0, M0 prostate cancer, and 99% for organ-confined cancer [1]. 

In lymph node positive prostate cancer after RP and adjuvant hormonal treatment 10-year CSS reaches 80% [2]. However, known lymph node metastasis remains a contraindication for most urologists for radical prostatectomy, and antihormonal treatment is initiated. Since then, the standing of radical prostatectomy as a treatment in this indication has been promoted by the findings of Engel et al., even in cases of suspected or proven lymph node metastasis. They were able to show that the survival of patients with lymph node metastasis was improved by radical prostatectomy when compared to patients who broke off surgery [3]. Adjuvant radiotherapy combined with hormonal treatment in lymph node involvement is advantageous when compared with hormonal treatment alone [4]. Preoperative prediction of lymph node involvement is challenging, especially in current-era prostate cancer with high percentage of low-risk prostate carcinoma that do not fit with the Roach formula, which overpredicts lymph node metastasis [5, 6]. 

We analysed a single-centre collective of patients suspected for ≥cT3 prostate carcinoma after radical prostatectomy and pelvic lymphadenectomy in order to establish the predictability of lymph node involvement by virtue of histopathological parameters.

## 2. Material and Methods

We retrospectively collected clinical and histopathological data of 34 patients who underwent RP for suspected ≥cT3 prostate cancer. Open surgery took place between 2007 and 2010 in a German single centre. 

Suspect digital rectal examination (DRE), elevated PSA level, lower urinary tract symptoms (LUTS) or hydronephrosis led to the suspicion of prostate cancer, respectively. Diagnosis was assessed by ultrasound-guided prostate biopsy. Locally advanced stage was indicated by suspect digital rectal examination and confirmed by computed tomography (CT). There was no evidence of lymph node involvement or organ metastasis in CT assessment. Before surgical therapy all patients underwent bone scans without detection of skeletal metastasis. To reduce local tumour mass, five patients were neoadjuvantly treated antihormonally.

We assessed pT and pN stage, the share of positive margins (R1) and compared bioptic and specimen Gleason scores as well as the predictive value of these parameters with regard to the existence of lymph node metastasis. 

## 3. Results and Discussion

### 3.1. Clinical and Histopathological Patient Data

34 patients with a median age of 65 years (range 55–75 years) and with suspected locally advanced prostate carcinoma had a median PSA level of 23 ng/mL (range 5–141 ng/mL) at the time of diagnosis. The day prior to surgery median PSA level was 25 ng/mL, but some patients only had one PSA testing run before surgery. Four out of five patients who underwent neoadjuvant antihormonal therapy had no further preoperative PSA testing; one patient's PSA level decreased from 98 to 2 ng/mL. 

The median Gleason score from the prostate biopsy was 8 (range 6–10) and from the prostatectomy specimen 9 [7–9]. Only in 44% Gleason score of prostate biopsy and specimen was identical; underestimation in prostate biopsy score of one to three points was detected in 41% of patients and overestimation of one score point in 15%. Patients showed pathological stage pT3a in 26%, pT3b in 59% and pT4 in 15%. For details see Table 1. Residual tumour defined by cancer positive margin of the prostatectomy specimen was found in 76%. Neoadjuvant treatment seemed to have a protective effect, with positive margins in 60% of these patients whilst patients without preoperative antihormonal therapy showed residual tumour in 79% of cases. Whilst 85% of patients with positive margins had lymph node metastasis, only 50% of the patients without residual prostate tumour mass showed lymph node involvement (Table 2). Median number of dissected lymph nodes was 15 (range 6–32), in the case of lymph node metastasis, and the median number of metastasis was 2 (range 1–10).

### 3.2. Discussion of Predictive Factors for the Existence of Lymph Node Metastasis

In a multicentre series of 712 patients, Spahn et al. showed that PSA levels >20 ng/mL were associated with organ-confined tumour in 33%, with Gleason score ≤6 in prostate biopsy in 8%, with negative surgical margins in 54%, and with no lymph node involvement in 85% of cases, respectively [7]. Patients with PSA levels >20 ng/mL and suspected locally advanced prostate cancer had positive margins in 79% and lymph node invasion in 51% of cases. Our results confirmed these findings by showing residual tumour and lymph node metastasis in 76% of cases. Using the same series, Gontero et al. found that the PSA level was of prognostic relevance with 26% cured by surgery alone when PSA was 20–50 ng/mL but only about 7–9% with PSA >50 ng/mL [8]. A single-centre analysis of more than 2 600 patients with locally advanced prostate cancer after RP and adjuvant androgen deprivation revealed the Gleason score to be the most important prognostic factor [9]. In our much smaller series we did not attempt to show cancer-specific survival, but for the prediction of T stage and lymph node involvement, the Gleason score was not the most obvious parameter. Evidence of residual tumour presence on the surgical margins in our patient collective was the most important predictive parameter for lymph node metastasis. 76% positive margins corresponded with 76% stage pN1. Patients with positive margins had synchronous lymph node metastasis in 85%, and negative margins were only associated in 50% with lymph node metastasization. Another study on a collective of high-risk prostate cancer (stage ≥pT3 in 89%) showed positive margins in 83% but only in 28% pN1 disease [10]. Oh et al. showed that positive margins in stage pT2 prostate cancer lead to a worse outcome, similar to that of patients with locally advanced prostate carcinoma [11].

## 4. Conclusions

Alongside the Gleason score and pathological T stage, the presence of positive surgical margins is an important predictive factor in estimating lymph node involvement. Neoadjuvant antihormonal therapy does lead to a relevant reduction in the rate of positive margins, but not to a reduction in the rate of lymph node metastasis. As such, antihormonal and surgical treatment should be considered in combination for the therapy of locally advanced prostate cancer.

## Figures and Tables

**Table 1:** Characteristics of patients with locally advanced prostate carcinoma.

No.	Age	PSA	GS biopsy	GS specimen	pT stage	pN stage
1	60	77,3	6	7	pT3b	pN1
2	66	36,0	6	7	pT3a	pN1
3	68	30,2	6	7	pT3a	pN1
4	62	58,0	6	8	pT3b	pN0
5	64	35,6	6	9	pT3a	pN0
6	59	60,0	6	9	pT3b	pN1
7	60	31,5	7	7	pT3b	pN1
8	65	52,0	7	7	pT3b	pN1
9	70	56,0	7	7	pT3b	pN1
10	74	17,8	7	8	pT3b	pN0
11	63	21,4	7	8	pT3b	pN1
12	75	14,2	7	9	pT3b	pN1
13	55	5,8	7	9	pT4	pN0
14	61	100,0	7	9	pT3a	pN0
15	63	14,1	7	9	pT4	pN1
16	62	47,0	8	7	pT3b	pN1
17	74	23,3	8	7	pT3a	pN1
18	63	73,0	8	8	pT3b	pN1
19	68	11,5	8	8	pT3a	pN0
20	71	15,2	8	8	pT3a	pN0
21	70	9,5	8	8	pT3b	pN1
22	71	141,0	8	9	pT3b	pN1
23	65	138,0	8	9	pT4	pN1
24	64	34,7	9	8	pT4	pN1
25	70	7,2	9	9	pT3a	pN1
26	69	14,6	9	9	pT3b	pN1
27	60	13,0	9	9	pT3b	pN1
28	70	100,0	9	9	pT3b	pN1
29	58	25,0	9	9	pT3a	pN0
30	67	100,0	9	9	pT3b	pN1
31	61	22,0	9	9	pT3b	pN1
32	68	2,2	9	9	pT3b	pN1
33	56	15,1	10	9	pT3b	pN1
34	70	15,0	10	9	pT4	pN1

GS: Gleason score.

**Table 2:** Association of positive margins with lymph node metastasis.

No.	Age	Neoadj. HT	R stage	pN stage
1	60	No	R1	pN1
2	66	No	R1	pN1
3	68	No	R1	pN1
4	62	No	R1	pN0
5	64	No	R0	pN0
6	59	No	R1	pN1
7	60	Yes	R0	pN1
8	65	No	R1	pN1
9	70	No	R1	pN1
10	74	No	R0	pN0
11	63	No	R1	pN1
12	75	No	R1	pN1
13	55	No	R1	pN0
14	61	Yes	R1	pN0
15	63	No	R1	pN1
16	62	Yes	R0	pN1
17	74	No	R0	pN1
18	63	No	R1	pN1
19	68	No	R0	pN0
20	71	No	R0	pN0
21	70	No	R1	pN1
22	71	No	R1	pN1
23	65	No	R1	pN1
24	64	No	R1	pN1
25	70	No	R0	pN1
26	69	No	R1	pN1
27	60	No	R1	pN1
28	70	Yes	R1	pN1
29	58	No	R1	pN0
30	67	No	R1	pN1
31	61	No	R1	pN1
32	68	Yes	R1	pN1
33	56	No	R1	pN1
34	70	No	R1	pN1

HT: antihormonal treatment.
